# Transcriptome analysis and phenotyping of walnut seedling roots under nitrogen stresses

**DOI:** 10.1038/s41598-022-14850-2

**Published:** 2022-07-14

**Authors:** Yan Song, Rui Zhang, Shan Gao, Zhiyong Pan, Zhongzhong Guo, Shangqi Yu, Yu Wang, Qiang Jin, Xiaofei Chen, Lei Zhang

**Affiliations:** 1 College of Plant Sciences, Tarim University, Alar, 843300 China; 2 National and Local Joint Engineering Laboratory for High-Efficiency and Quality Cultivation and Deep Processing Technology of Characteristic Fruit Trees in Southern Xinjiang, Alar, 843300 China; 3 Key Laboratory of Protection and Utilization of Biological Resources in Tarim Basin, Tarim University, Alar, 843300 China; 4 College of Life Sciences, Tarim University, Alar, 843300 China

**Keywords:** Molecular biology, Physiology, Plant sciences

## Abstract

Nitrogen is an essential core element in walnut seedling growth and development. However, nitrogen starvation and excessive nitrogen stress can cause stunted growth and development of walnut seedlings, and environmental pollution is also of concern. Therefore, it is necessary to study the mechanism of walnut seedling resistance to nitrogen stress. In this study, morphological and physiological observations and transcriptome sequencing of walnut seedlings under nitrogen starvation and excess nitrogen stress were performed. The results showed that walnut seedlings under nitrogen starvation and excess stress could adapt to the changes in the nitrogen environment by changing the coordination of their root morphology and physiological indexes. Based on an analysis of transcriptome data, 4911 differential genes (DEGs) were obtained (2180 were upregulated and 2731 were downregulated) in a comparison of nitrogen starvation and control groups. A total of 9497 DEGs (5091 upregulated and 4406 downregulated) were obtained in the comparison between the nitrogen overdose and control groups. When these DEGs were analysed, the differential genes in both groups were found to be significantly enriched in the plant’s circadian pathway. Therefore, we selected the circadian rhythm as the focus for further analysis. We made some discoveries by analysing the gene co-expression network of nitrogen metabolism, circadian rhythm, and hormone signal transduction. (a) Nitrite nitrogen (NO_2_^−^) or Glu may act as a nitrogen signal to the circadian clock. (b) Nitrogen signalling may be input into the circadian clock by regulating changes in the abundance of the CRY1 gene. (c) After the nitrogen signal enters the circadian clock, the expression of the LHY gene is upregulated, which causes a phase shift in the circadian clock. (d) The RVE protein may send information about the circadian clock’s response to nitrogen stress back to the nitrogen metabolic pathway via the hormone transduction pathway. In conclusion, various metabolic pathways in the roots of walnut seedlings coordinated with one another to resist the ill effects of nitrogen stress on the root cells, and these coordination relationships were regulated by the circadian clock. This study is expected to provide valuable information on the circadian clock regulation of plant resistance to nitrogen stress.

## Introduction

The walnut (*Juglans regia* L.) is an economically important woody plant throughout the world^1^. China is one of the world’s largest walnut producers, with more than 40% of the global cultivated area^2^. The Xinjiang Uygur Autonomous Region is one of the primary production areas for walnut cultivation in China. Although its cultivated area covers only approximately 5% of the country, its output ranks as the second-highest in China^3^. Walnut planting has become an important industry for agricultural economic development and farmer prosperity in Xinjiang^4^. Despite the rapid development of the walnut industry in Xinjiang in recent years, the lack of standardised fertilisation management has largely restricted improvements in walnut quality. Our team’s previous study found that too little or too much nitrogen due to improper fertilisation would affect the normal growth of walnut plants and nut quality. Too little nitrogen would result in insufficient growth and reduced stress resistance in walnuts. High nitrogen application can lead to barren walnut branches, can aggravate winter freezing damage, and can further accelerate the invasion of walnut trees by of pests and diseases^5,6^. Therefore, it is of great significance to study the responses of walnut plants to nitrogen stress and to promote the establishment of a standardised fertilisation model for this crop.

Some advances have been made in understanding the mechanism of plant responses to nitrogen stress^7,8^. When the supply of exogenous nitrogen is insufficient, the growth behaviour of plants is abnormal. For example, leaf yellowing^9^, decreased lodging resistance^10^, and decreased quality and yield^11^ were observed. When the nitrogen supply exceeds the nitrogen loading capacity of plants, the concentrations of ascorbic acid and titratable acid in the fruits will increase^12^, leading to lower fruit quality. In addition, excessive nitrogen is often lost through rainwater erosion, denitrification, and volatilisation and other ways^13^. Part of the lost nitrogen infiltrates into surface water and groundwater, causing the eutrophication of water bodies and a series of environmental problems^14^. Therefore, it is very important to study the responses of plants to nitrogen stress. The plant circadian clock help plants withstand different types of environmental stress^15^. Gutiérrez et al*.*^16^ found that ChIP assays in both wild-type and CCA1-ox lines were able to confirm binding of CCA1 to the promoter regions of GLN1.3, GDH1 promoters. This conclusion proves that the Regulatory Role of CCA1 in the N-Assimilatory Pathway. And at the same time, they provided pulses of inorganic or organic N at intervals spanning a circadian cycle and determined the effects on the phase of the oscillation in CCA1::LUC expression. Each treatment resulted in stable phase shifts indicating that N status serves as an input to the circadian clock. Zhou et al*.*^17^ found that nitrogen affected the phosphorylation and abundance of plant cryptochrome (CRY), indicating that CRY acted on the nitrogen input pathway of the circadian clock. However, only a few studies have noted the relationship between nitrogen stress and the circadian clock, and it has not been reported in walnut studies.

In view of this context, this study used “Xincuifeng” walnut seedlings as the test material to determine the root morphology and some physiological indexes of walnut seedlings under nitrogen stress (nitrogen starvation and excess) and to analyse the transcriptome differences in walnut seedling root systems to explore the mechanism of walnut seedling resistance to nitrogen stress. The aim was to provide a theoretical basis for formulating a scientific fertilisation strategy and efficient nutrient management for walnut production.

## Results

### Morphology and physiology

The root morphology and physiology of walnut seedlings under different nitrogen stress treatments were significantly different (Table 1 and Supplementary Table S1). The results showed that the root fractal dimension under nitrogen starvation (EL) and nitrogen excess (H) stress was significantly (*P* < 0.05, the same below) higher than that of the control (L), at 1.03 times and 1.06 times, respectively. The nitrate nitrogen, ammonium nitrogen, and total nitrogen contents of the roots of walnut seedlings under N starvation stress were significantly lower than those of the control, and the protein and total amino acid contents were 3.89% and 9.26% higher than those of the control, respectively. The contents of zeatin (ZA), indole acetic acid (IAA), abscisic acid (ABA) and gibberellin (GA3) under nitrogen excess stress were significantly higher than those of the control. Under nitrogen starvation stress, the IAA and GA3 contents were significantly higher than those of the control, while the ZA was significantly lower than that of the control (Supplementary Table S1).

**Table 1:** Nitrogen starvation and excess stress effects on the root morphology and physiology of walnut seedlings.

Treatment	Surface area	Volume	Fractal dimension	Proline content	Malondialdehyde content	Total nitrogen content
	Cm^2^	Cm^3^		µg/g	nmol/g	g/kg
EL	460.90 ± 8.2024 a	67.38 ± 3.0710 a	1.61 ± 0.0055 a	435.38 ± 3.6660 b	27.28 ± 0.2345 b	26.66 ± 0.3959 b
L	274.44 ± 2.8425 c	36.67 ± 0.8851 b	1.56 ± 0.0203 b	419.81 ± 5.7013 b	25.14 ± 0.2022 c	29.40 ± 0.5243 a
H	360.52 ± 7.9209 b	68.10 ± 2.5963 a	1.65 ± 0.0057 a	490.90 ± 5.1761 a	28.21 ± 0.0517 a	30.24 ± 0.6002 a

The first column lists the different treatments (L = control, EL = nitrogen starvation, H = nitrogen excess), while the top row shows the indicators and the second row shows the indicator units. The data are the means ± SE. Different letters indicate statistical significance between the treatments tested by the analysis of variance (P < 0.05).

### Summary of RNA sequencing results

Total RNA was extracted from the roots of walnut seedlings treated with different levels of nitrogen (L, EL, and H). Each treatment had three biological replicates, and a total of nine cDNA libraries were constructed (Supplementary Table S2). Sequencing was performed on the BGISEQ-500 platform. The three treatments yielded a total of 71.81 million (EL), 69.96 million (L), and 69.97 million (H) raw reads, from which 67.47 million, 65.74, million and 66.58 million clean reads were obtained, respectively. After filtering, 97.43%, 97.34%, and 97.58% of the bases had a quality score > 20. The results showed that the quality of RNA-seq was satisfactory and met the requirements for data analysis.

### Differentially expressed gene analysis

**Figure 1:**
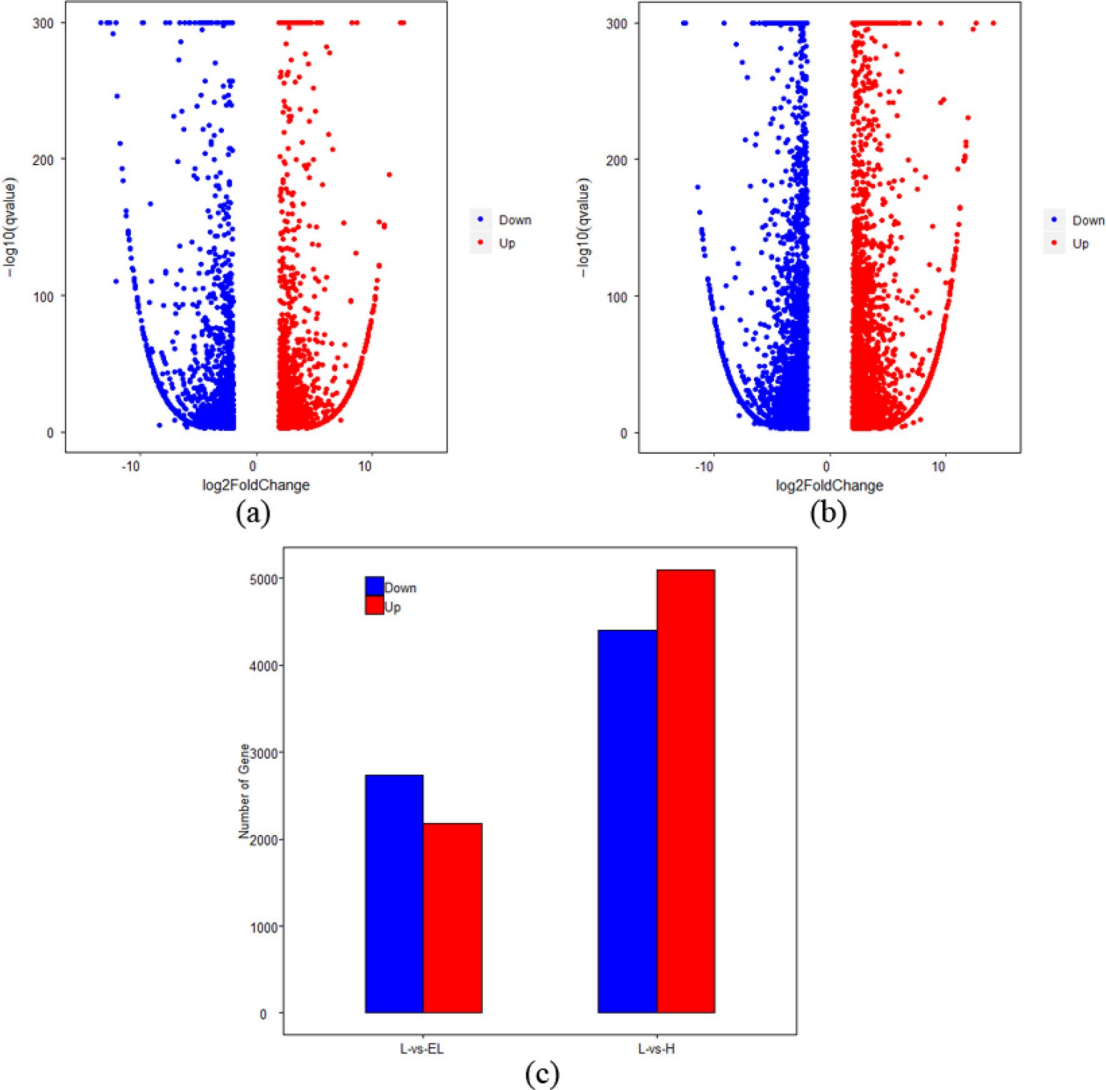
Volcano plots of DEGs in the roots of walnut seedlings under nitrogen starvation (**a**) and excess (**b**). Red dots indicate genes upregulated in the treatment group compared to control, and blue dots indicate genes downregulated in the treatment group relative to control. (**c**) Numbers of up- and downregulated genes under nitrogen starvation versus control (L-vs-EL) and under nitrogen excess versus control (L-vs-H). Red bar: number of upregulated genes; blue bar: number of downregulated genes.

We mapped the clean reads to the walnut genome sequence using Bowtie2 and then calculated the gene expression levels in each sample with RSEM software. Statistical analysis was performed using the DESeq R package to obtain DEGs from two comparisons: control versus nitrogen starvation (L-vs-EL) and control versus nitrogen excess (L-vs-H). DEGs were those that had a *q*-value < 0.001 and log2(fold change) > 2. In the L-vs-EL group, a total of 4911 DEGs were identified, 2180 upregulated and 2731 downregulated. In the L-vs-H group, a total of 9497 DEGs were identified, 5091 upregulated and 4406 downregulated (Fig. 1).

### Gene ontology (GO) enrichment analysis of DEGs

**Figure 2:**
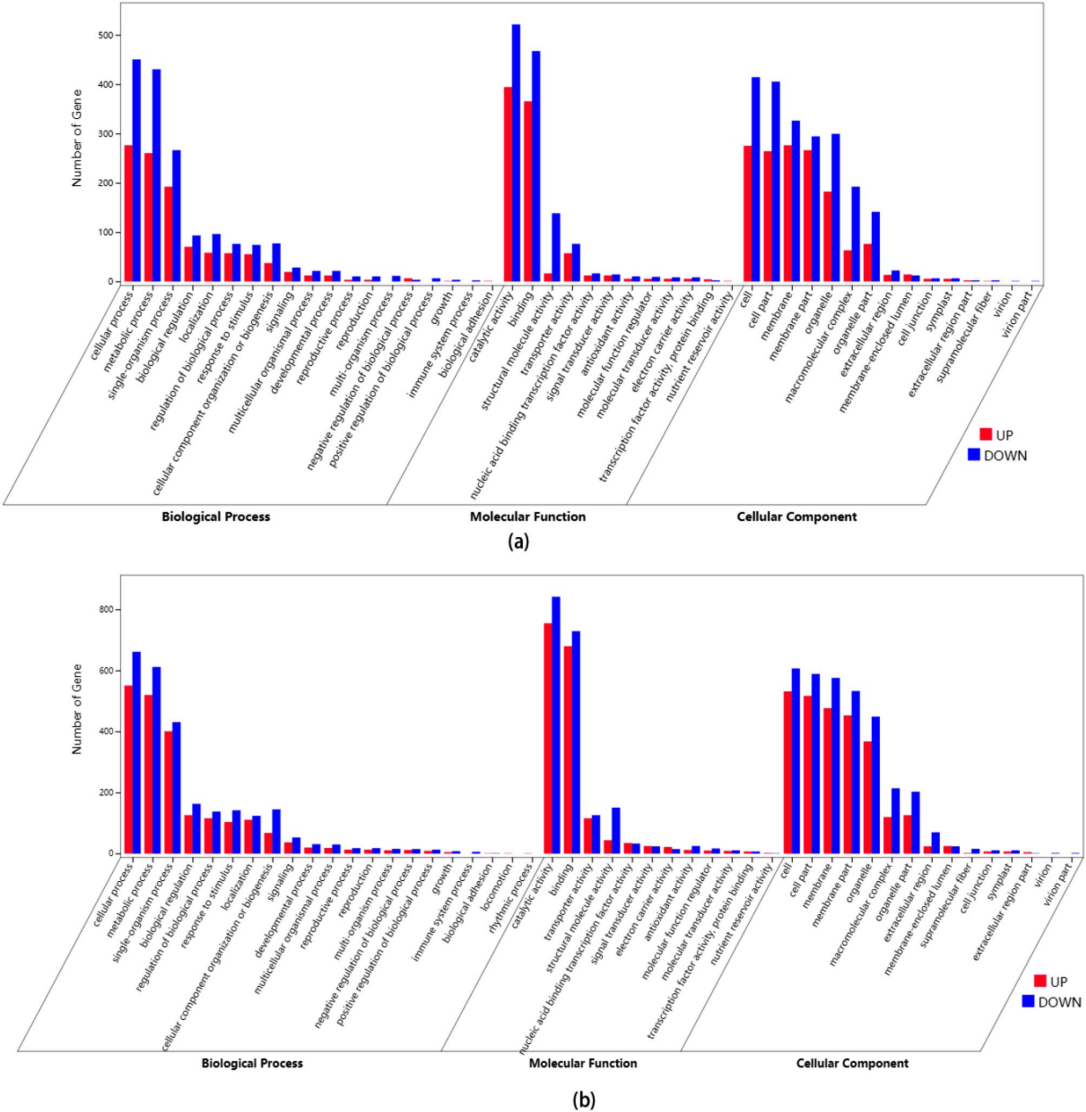
Gene ontology (GO) annotation of differentially expressed genes in the roots of walnut seedlings under nitrogen starvation (**a**) and excess (**b**). The ordinate represents the number of genes, and the abscissa represents the GO terms. Red bar: number of upregulated genes; blue bar: number of downregulated genes.

Through GO enrichment analysis, the DEGs of the L-vs-EL and L-vs-H groups were classified into three categories, namely, biological processes, molecular functions, and cellular components (Fig. 2). Each category is further divided into 46 subcategories and 48 subcategories. In general, the DEGs of walnut seedlings under N starvation and excess N stress were highly enriched in cellular processes, metabolic processes, single-organism processes, catalytic activity, binding, cells, cell parts and membranes. These results indicated that the root systems of the walnut seedlings regulated root cell damage through the coordination of multiple biological processes, and most of these interactions occurred on the cell membrane and in some cells.

### Kyoto encyclopedia of genes and genomes (KEGG) enrichment analysis of DEGs

**Figure 3:**
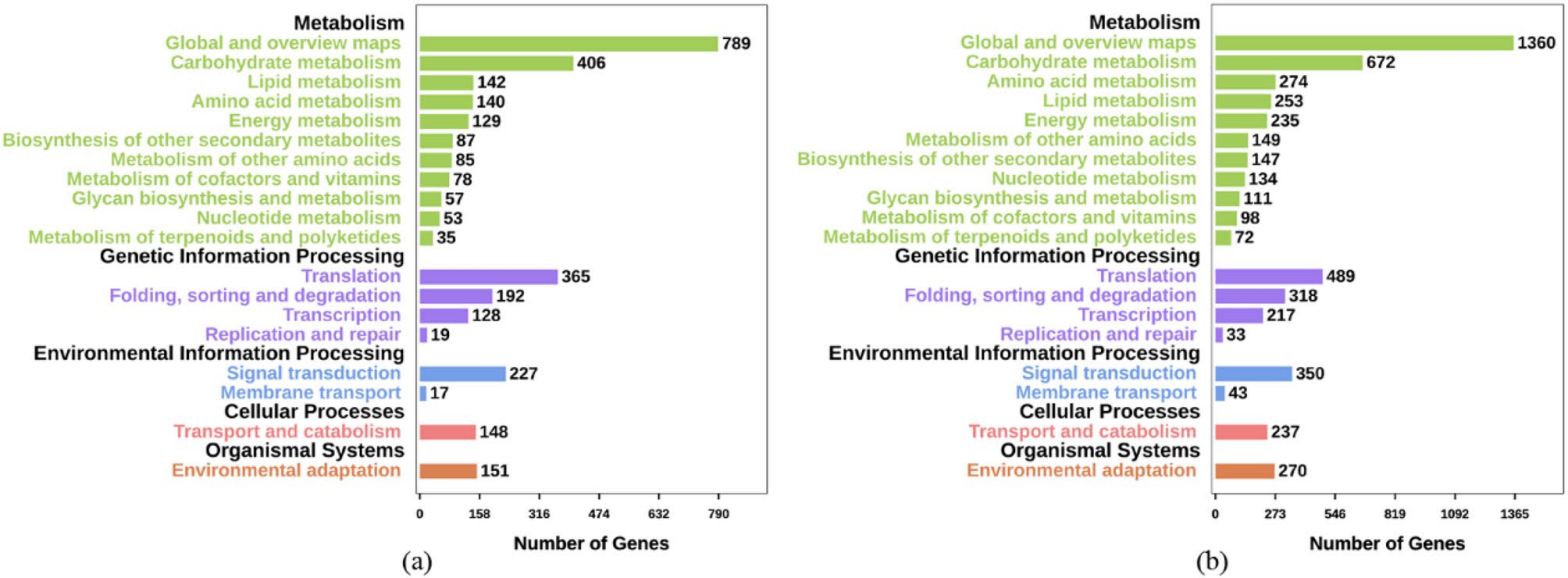
KEGG annotation of DEGs in the roots of walnut seedlings under nitrogen starvation (**a**) and excess (**b**). The ordinate represents the KEGG pathway, and the abscissa represents the number of genes.

To explore the metabolic regulatory pathways in the roots of walnut seedlings under nitrogen starvation and excess, we performed KEGG enrichment analysis on the DEGs in the two pairwise comparisons using the KEGG database (Fig. 3). We found that the DEGs of the two pairwise groups were divided into five KEGG classes (metabolism, genetic information processing, environmental information processing, cellular processes, and organismal systems; Fig. 3). The DEGs of L-vs-EL were enriched in 125 pathways, with significant enrichment in 19 pathways (*p* < 0.05), such as circadian rhythm-plant, carbon metabolism, and starch and sucrose metabolism. The DEGs of L-vs-H were enriched to 133 pathways, with significant enrichment in 28 pathways (*p* < 0.05), such as nitrogen metabolism, circadian rhythm-plant, carbon metabolism, and starch and sucrose metabolism.

### Co-expression network analysis of genes related to nitrogen metabolism, circadian rhythm and plant hormone signal transduction

**Figure 4:**
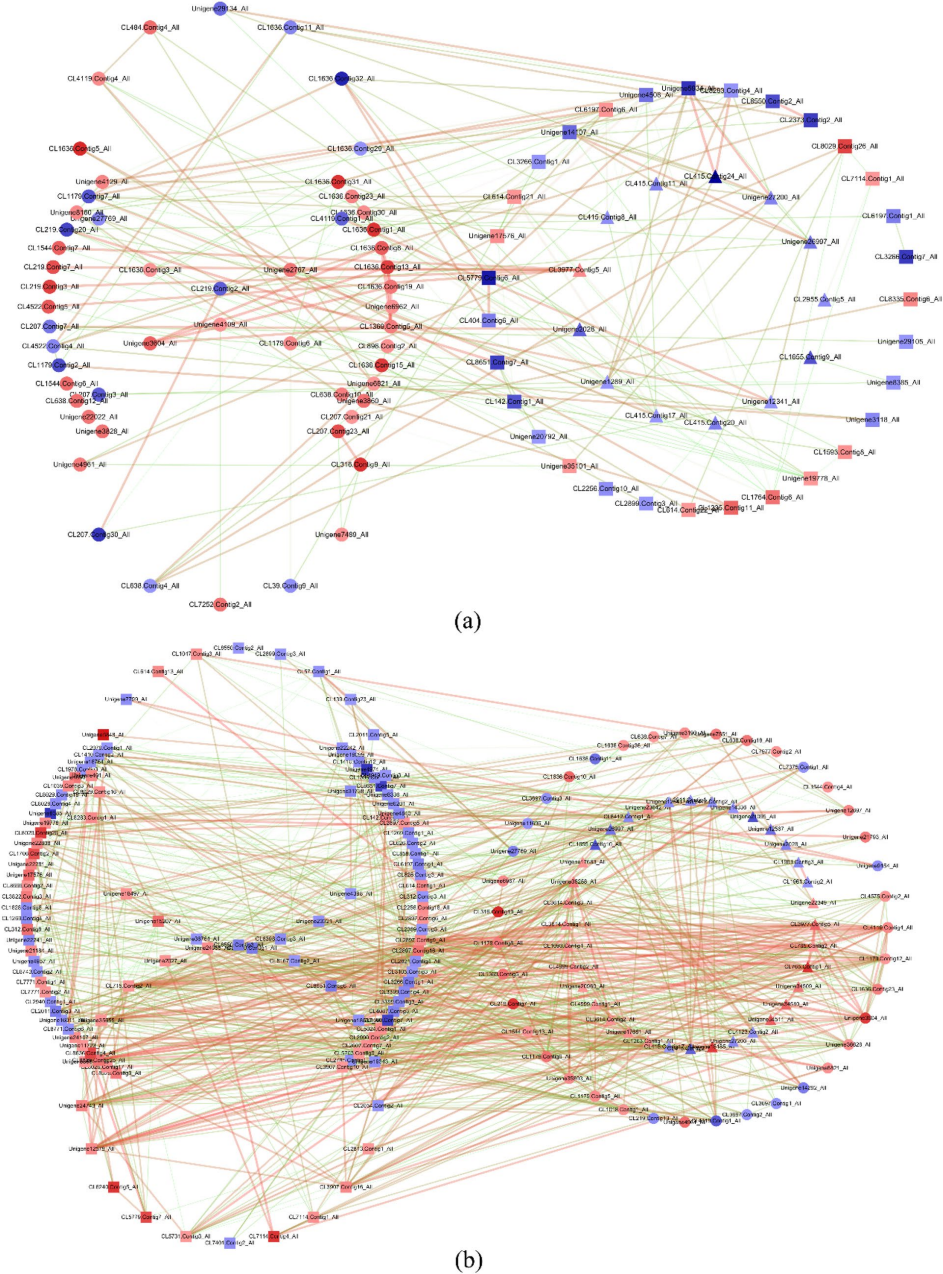
Gene co-expression networks of nitrogen metabolism, circadian rhythms, and hormone signal transduction pathways in the root system of walnut seedlings under nitrogen starvation (**a**) and excess (**b**). **○** represents genes related to circadian rhythms, **△** represents genes related to nitrogen metabolism, and **□** represents genes related to plant hormone signal transduction. The colour gradient of connections, from green to red, indicates a Pearson correlation coefficient from −1 to 1; a darker colour and a thicker connection indicate a higher correlation. The colour gradient of dots, from blue to red, indicates a log_2_(fold change) from negative to positive, and a darker colour indicates a greater absolute value of log_2_(fold change).

A KEGG analysis showed that DEGs in both comparison groups were significantly enriched in the plant circadian pathway. Moreover, nitrogen metabolic pathways and hormone signal transduction may play an important role in plant responses to extreme nitrogen conditions. Therefore, we constructed the regulatory network relationships among the root nitrogen metabolism, circadian rhythm, and hormone signal transduction of walnut seedlings (Fig. 4). We selected 106 DEGs from the L-vs-EL comparison and 191 DEGs from the L-vs-H comparison to construct gene co-expression networks. The numbers of selected genes related to nitrogen metabolism, circadian rhythms, and hormone signal transduction were 15, 59, and 32 (L-vs-EL) and 37, 40, and 114 (L-vs-H), respectively. The nodes of each network were gene combinations whose Pearson correlation coefficients had an absolute value > 0.95 (Fig. 4). We found that the network of L-vs-EL contained 98 nodes and 226 connections, with 188 pairs of genes being positively correlated and 38 pairs of genes being negatively correlated. The network of L-vs-H contained 189 nodes and 971 connections, with 898 pairs of genes being positively correlated and 74 pairs of genes being negatively correlated.

### Validating the expression patterns of selected DEGs by qRT-PCR

**Figure 5:**
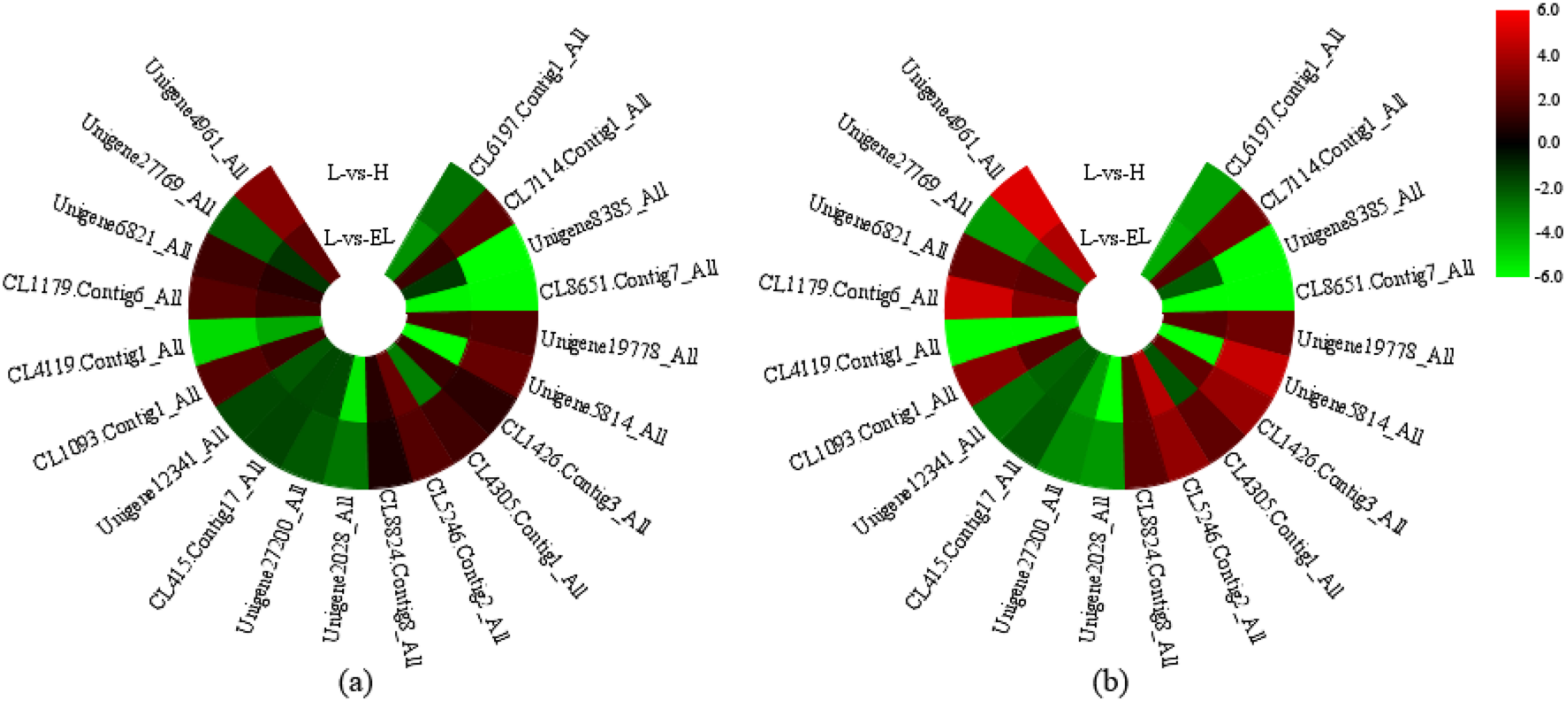
Expression levels of 20 differentially expressed genes obtained by qRT-PCR; (**a**) were used to validate the RNA-seq data (**b**). Both the qRT-PCR and the RNA-seq data are the mean values of three biological replicates. The colour gradient from green to red indicates low to high gene expression. H, EL, and L represent nitrogen excess, nitrogen starvation, and control treatment, respectively.

To verify the reliability of the RNA-seq data, we selected 20 DEGs involved in the nitrogen metabolism pathway (5), circadian rhythm pathway (5), hormone signal transduction pathway (5), and other genes (5). Their expression levels after nitrogen excess (H), nitrogen starvation (EL), and control (L) treatments were analysed by qRT-PCR. There was good consistency between the qRT-PCR and RNA-seq data for all 20 genes (Fig. 5), indicating that the RNA-seq data were reliable.

## Discussion

### Morphological and physiological responses to nitrogen stress

Excess nitrogen and nitrogen starvation stress had significant effects on the root growth of the walnut seedlings. The fractal dimension provides an accurate estimate of the plant root growth Berntson et al*.*^18^. The results showed that the root fractal dimension under nitrogen excess and nitrogen starvation stresses was significantly higher than that of the control. Hu et al*.*^19^ found that wheat under nitrogen stress and water stress could improve its adaptability to the stress environment by changing its root morphology. These results indicated that the root system of walnut seedlings under nitrogen starvation could better capture nitrogen in the environment by promoting the growth of the root system. However, excessive nitrogen stress resulted in the accumulation of nitrogen in the roots, so there was an urgent need to promote the growth of roots to absorb water from the environment and adjust the balance of water and nitrogen in the roots.

Amino acids and proteins are known essential nutrients for plant body weight and constitute various physiologically active substances in plants. In this study, excessive nitrogen stress promoted the synthesis of total amino acids and proteins. However, the total amino acid and protein contents in roots under N starvation stress were higher than those of the control. Based on previous studies, nitrogen starvation can also induce the accumulation of phenylalanine ammonia lyase (PAL). PAL removes NH_4_^+^ from phenylalanine, while NH_4_^+^ participates in amino acid metabolism after assimilation^20–22^. This may be a stress mechanism of the root response to nitrogen starvation stress. The malondialdehyde (MDA) and proline (Pro) contents increased significantly under nitrogen stress. In addition, the MDA and Pro contents under nitrogen overdose stress were significantly higher than those under nitrogen starvation stress. The results indicated that the cell damage in walnut seedling roots caused by excessive nitrogen stress was more serious than that caused by nitrogen starvation stress. Plant hormones can improve plant resistance to abiotic stress by regulating root growth^23^. When plants were faced with a nitrogen stress challenge, the IAA and GA3 contents were significantly increased, which was consistent with the results of Li et al*.*^24^ and Lv et al*.*^25^. It could be that walnut seedlings under nitrogen starvation need to synthesize large amounts of GA3 to promote root elongation and better absorb nitrogen from the soil. The NO_3_^-^ concentration in roots can promote the rapid increase of the cytokinin concentration^26^, while cytokinin inhibits NO_3_^-^ uptake and nitrate transporter (NRT) synthesis in plants under nitrogen excess stress, thus slowing down nitrogen uptake^27^. ZA is a natural cytokinin found in plants. The results showed that the ZA content in the roots of walnut seedlings under N starvation stress was significantly lower than that of the control, while the ZA content under excess N stress was significantly higher than that of the control. These results indicated that ZA could regulate N uptake by walnut seedling roots under N starvation and N excess stress.

In conclusion, in the face of nitrogen starvation and excess nitrogen stress, the walnut seedling root system can improve the adaptability of walnut seedlings to nitrogen starvation and excess nitrogen stress through morphological regulation and the coordination of physiological indexes.

### Circadian clock controls walnut seedling resistance to nitrogen stress

GO and KEGG enrichment analyses of DEGs from the RNA-Seq data of the nitrogen starvation comparison group (L-VS-EL) and nitrogen excess comparison group (L-VS-H) showed that the genes involved in plant circadian rhythm regulation were significantly different from those of the control group under both nitrogen starvation and excess nitrogen stress. Circadian rhythms are an endogenous timing mechanism of plants and a manifestation of the circadian clock. A stimulus signal in the environment is transmitted to the core oscillator through the input pathway, and the oscillator is the cell part that responds to environmental stimuli; the response signal is passed through the output pathway to the corresponding biological process, thereby controlling plant responses to stimuli^28^. CRY is a key gene in the circadian clock input pathway, and the *Arabidopsis* CRY1 gene, located in the nucleus^29^, primarily mediates blue light to regulate plant desolation^30^. Zhou et al*.*^17^ found that low nitrogen treatment increased the ratios of NADPH/NADP^+^ and ATP/AMP and affected the phosphorylation and abundance of CRY1, thus triggering a phase shift in the circadian clock. In this study, we found that excess nitrogen stress promoted the expression of the CRY1 gene. These results indicated that the phase shift of the circadian clock in walnut seedlings was caused by excess nitrogen stress, and the CRY1 protein may be an important key to nitrogen signal input into the circadian clock. Given an analysis of the gene co-expression network, the expression of the CRY1 gene was significantly positively correlated with the ferredoxin-nitrite reductase (Fd-NIR) gene and the ferredoxin-dependent glutamate synthase (Fd-GOGAT) gene. We speculate that the nitrogen metabolites nitrite nitrogen (NO_2_^-^) or Glu may act as nitrogen signals to input into the circadian clock. This finding adds to the conclusion that nitrogen metabolites may be an input signal of the circadian clock^16^.

Late elongated hypocotyl (LHY) and CCA1 are MYB transcription factors with high coding homology and partial functional redundancy^31^. Both are important components of the core oscillator of the circadian clock^32^. Gutiérrez et al*.*^16^ found that the expression of CCA1 could be regulated by the nitrogen metabolites Gln and Glu. In this study, the expression of the LHY gene was upregulated under nitrogen overstress. This finding indicates that the nitrogen signal input to the circadian clock through CRY1 controls the entire core oscillator, resulting in a phase shift of the circadian clock. Yang and Midmore^33^ found that the gene expression of nitrate reductase (NR) in most higher plants had diurnal variation. In this study, through the analysis of a gene co-expression network, the expression of the NR gene was found to be significantly positively correlated with the expression of the LHY gene. It was speculated that the NR gene expression in the root system of walnut seedlings was positively regulated by the LHY gene, which resulted in the diurnal variation of the NR gene in the root systems of walnut seedlings.

The Reveille (RVE) gene has homology with CCA1 and LHY genes, but it only participates in the clock output of plant development^34,35^. In this study, we found that nitrogen stress affects the expression of the RVE gene, and the RVE gene may promote the expression of the auxin transporter-like PROTEIN 2 (LAX2) gene. This finding suggests that the RVE gene, the “messenger” of the circadian clock, carries the information needed to address nitrogen stress through the hormone transduction pathway to the nitrogen metabolism pathway.

## Conclusions

**Figure 6:**
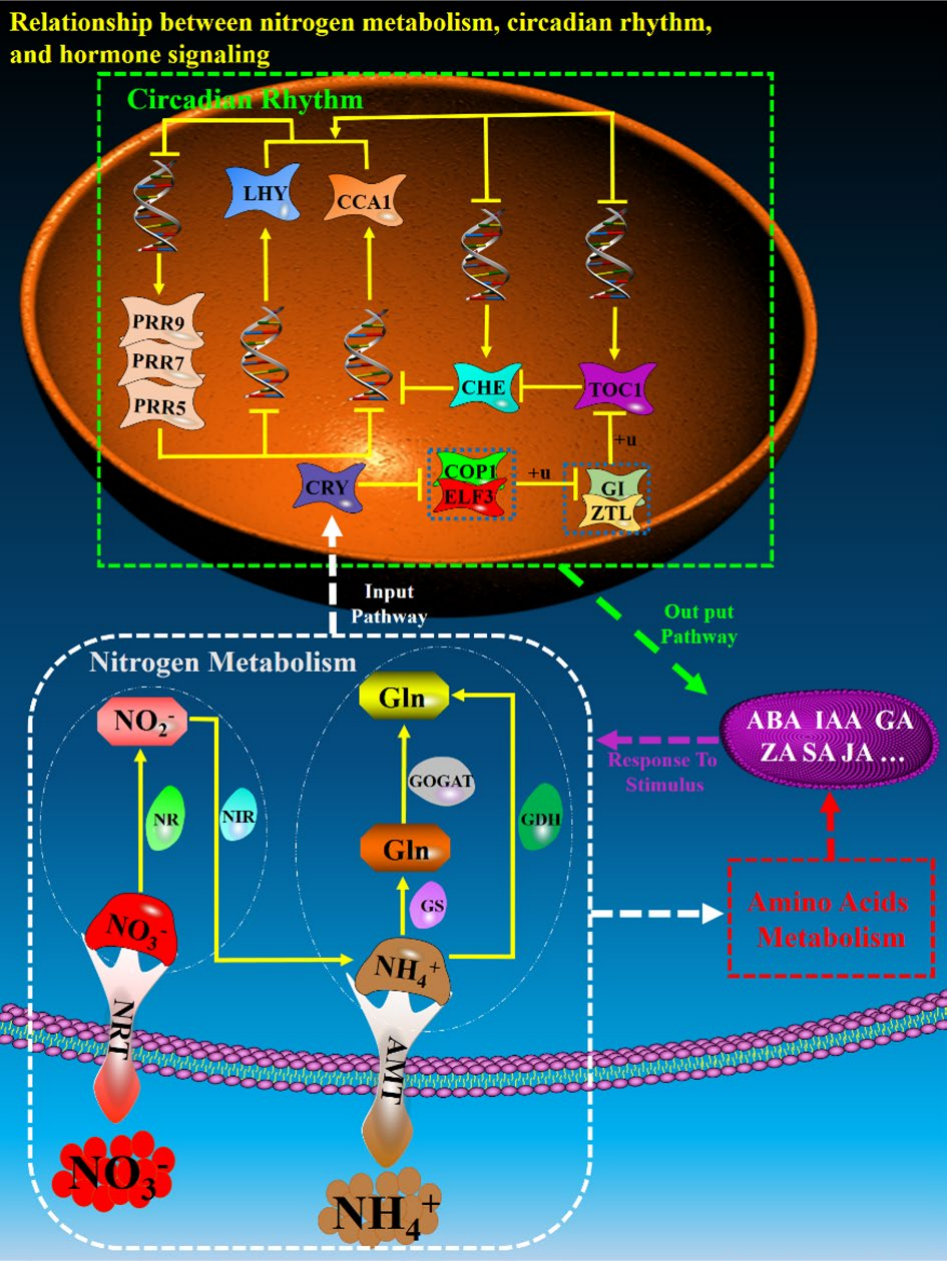
Hypothetical model showing the mutual regulation of nitrogen metabolism, circadian rhythms, and hormone signal transduction. The combination of two rows of purple balls with yellow connections between them represents the cell membrane, and the orange ellipse represents the cell nucleus. Dashed arrows, solid arrows, and T-shaped segments represent possible input and output pathways, activation, and inhibition, respectively. The thin white dotted line is used to distinguish NO_3_^-^ reduction and NH_4_^+^ assimilation in nitrogen metabolism, while the blue dotted boxes in the circadian rhythm pathway represent protein complexes. The white, green, and red thick dotted boxes and the purple ellipse represent nitrogen metabolism, circadian rhythms, amino acid metabolism, and hormone signal transduction pathways, respectively. From bottom to top, the cluster of red and brown balls represents NO_3_^-^ and NH_4_^+^ outside the cell, respectively; the ‘tree’-shaped molecules represent the transmembrane transporters NRT and AMT; the ‘caps’ on the transmembrane proteins represent NO_3_^-^ and NH_4_^+^ transported into the cell; hexagons with different colours represent various products of nitrogen metabolism; in the nitrogen metabolism pathway, irregular shapes with different colours attached to each line represent different enzymes of nitrogen metabolism; in the circadian rhythm pathway, irregular shapes with different colours represent different proteins; the double helix structure represents DNA; and the ‘ + u’ around the solid line represents ubiquitination.

In conclusion, various metabolic pathways in the roots of walnut seedlings are coordinated to resist the ill effects of nitrogen stress on root cells, and these coordinated relationships are regulated by the circadian clock. Therefore, we plotted the "nitrogen metabolism-plant circadian cycle network regulation mode (NCRM)" based on the "Arabidopsis circadian clock"^36^. (Fig. 6). In nitrogen metabolites, the free nitrite (NO_2_^−^) signal or Glu signal enters the nucleus to promote the expression of the CRY gene, which causes a phase shift in the circadian clock and produces response information to nitrogen stress. The response information is then transmitted to the nitrogen metabolism pathway through the hormone signal transduction network to control the activity of key enzymes in nitrogen metabolism, reduce the accumulation of NO_3_^-^ and NH_4_^+^, and reduce cell damage.

## Methods

### Plant materials and simulation of nitrogen stresses

The experiment was performed in a greenhouse at the College of Plant Science, Tarim University, Alar, Xinjiang, China. Seeds from the walnut (*Juglans regia* L.) cultivar “Xincuifeng” of similar sizes, weights, and shapes were selected. The walnut seeds used in this study comply with the IUCN Policy Statement on Research Involving Species at Risk of Extinction and the Convention on the Trade in Endangered Species of Wild Fauna and Flora. The seeds were soaked in running water for a week and then exposed to the sun until they cracked at the suture line. The seeds were sown in 25-L containers filled with substrate (perlite: vermiculite: peat soil = 5:4:1). After the development of two pinnately compound leaves, seedlings with consistent growth were retained for later nitrogen treatment. CO(NH_2_)_2_ was used as the nitrogen source to formulate a 0.05 mol/L nitrogen-containing aqueous solution as the control (L), while 0 mol/L and 0.25 mol/L nitrogen-containing aqueous solutions were used for nitrogen starvation (EL) and excess (H) treatments. Samples were taken after 7 days of treatment, with three biological replicates per treatment. The roots were cleaned immediately, and root data were rapidly collected using a root scanner. The roots were separated and frozen in liquid nitrogen, then stored at − 80 °C before RNA extraction and physiological measurements.

### Measurement of root morphological and physiological data

The total lengths, surface areas, volumes, average diameters, and fractal dimensions of the roots of the walnut seedlings were measured using a plant root scanner (Wanshen LA-S, China). The total root length refers to the sum of the lengths of the main root, lateral roots, and fibrous roots. The physiological indexes we determined included the Pro, MDA, total nitrogen, protein, total amino acid, nitrate nitrogen, ammonium nitrogen, zeatin, indoleacetic acid, abscisic acid and gibberellic acid contents. The methods used to determine these physiological indexes are listed in Annex 1.

### Total RNA extraction, cDNA library construction, and sequencing

The total RNA was extracted using the cetyltrimethylammonium bromide (CTAB) method with an E.Z.N.A. Plant RNA Kit (Omega Bio-Tek, USA), with three biological replicates per treatment (EL1, EL2, EL3, L1, L2, L3, H1, H2, and H3). The RNA concentration was measured using the NanoDrop One (Thermo Fisher Scientific, USA). RNA degradation and contamination were checked by 1% agarose gel electrophoresis. The mRNA with poly-A tails was enriched using magnetic oligo-dT beads, and the resulting RNA was fragmented with fragmentation buffer and reverse-transcribed with random N6 primers. Single cDNA strands were synthesised to form double-stranded DNA. Thereafter, the synthesised double-stranded DNA was blunted and phosphorylated at the 5′-end. In addition, a cohesive end with a protruding “A” was formed at the 3′-end, which was ligated to a blister-like linker with a protruding “T” at the 3′-end. The ligation product was amplified by PCR with specific primers. The PCR product was thermally denatured into single strands. The single-stranded DNA was circularised with bridge primer to obtain a single-stranded circular DNA library. The constructed sequencing library was sequenced on the BGISEQ-500 platform (BGI, China). The construction and sequencing of cDNA libraries was completed by BGI in Shenzhen, China.

### Sequence analysis

The raw reads were counted using SOAPnuke filtering software (Shenzhen Huada Gene Research Institute, China) independently developed by BGI and were filtered using Trimmomatic (THE USADEL LAB, USA). The clean reads were aligned to reference gene sequences using Bowtie2^37^. Then, the gene expression levels were calculated using RSEM^38^. A statistical analysis was performed using the DESeq R package^39,40^. DEGs were obtained using L-vs-EL and L-vs-H comparisons.

### GO and KEGG enrichment analysis of DEGs

The DEGs were classified using the Gene Ontology (GO) (www.geneontology.org)^41^ and Kyoto Encyclopedia of Genes and Genomes (KEGG) (www.kegg.jp)^42^ databases to obtain GO- and KEGG-annotated background genes. Those DEGs with a *q*-value < 0.001 and log_2_(fold change) > 2 were selected. The GO- and KEGG-annotated background gene files and the selected DEGs were used for GO and KEGG functional enrichment analysis and data visualisation in OmicShare (www.omicshare.com).

### Construction of gene co-expression networks for nitrogen metabolism, circadian rhythms, and hormone signal transduction

To explore the relationships among nitrogen metabolism, circadian rhythms, and hormone signal transduction, a gene co-expression network analysis was performed. In accordance with the method of Khadka et al*.*^43^, the expression data of nitrogen metabolism, circadian rhythm, and hormone signal transduction genes were processed. Then, a cut-off value of *P* > 0.95 was set to select the data. The selected co-expression network data were visualised in Cytoscape (Cytoscape, USA). Finally, the key genes in the gene co-expression network were identified at degree > 10.

### Validation of RNA-seq by qRT-PCR

The reliability of the RNA-seq data was validated by qRT-PCR. The total RNA of 20 candidate genes was extracted using the CTAB method and an E.Z.N.A. Plant RNA Kit (Omega Bio-Tek), with three biological replicates per treatment. The first-strand cDNA was synthesised using EasyScript One-Step gDNA Removal and cDNA Synthesis Super Mix kit (Beijing TransGen Biotech Co., Ltd., China). The primers were designed using the Primer-BLAST (www.ncbi.nlm.nih.gov/tools/primer-blast/) tool from the National Centre for Biotechnology Information database (Supplementary Table S3). With the 18S rRNA gene as the reference control, the expression of each gene relative to the 18S rRNA gene was calculated using the 2^-△△Ct^ method. The PCR program was as follows: step 1 (pre-denaturation): 95 °C, 2 min; step 2 (PCR amplification; 45 cycles): 95 °C, 15 s; T_m_ value, 15 s; 55 °C, 20 s; and step 3 (melting curve): 95 °C, 15 s; 60 °C, 15 s; δ, 20 min; and 95 °C, 15 s. The fluorescent dye used for the reactions was TB GreenTM Premix Ex TaqTM II (Tli RNase H Plus) RR820A kit (TaKaRa, Japan). The qRT-PCR was run in an Eppendorf Realplex 2 fluorescent quantitative PCR system (Eppendorf, Germany).

### Statistical analysis and visualisation of data

The root morphological and physiological data were subjected to the least significant difference test, and the differences were considered significant at *P* < 0.05. The Pearson’s correlation coefficients between the genes related to nitrogen metabolism, circadian rhythms, and hormone signal transduction were calculated. Both analyses were performed using IBM SPSS Statistics 20 (International Business Machines Corporation, USA). In addition, volcano plots and histograms for the selected DEGs were created using the ggplot2 R package^44^. A hypothetical model for the mutual regulation of nitrogen metabolism-circadian rhythm-hormone signal transduction was drawn in Microsoft Office PowerPoint 2013 (Microsoft, USA).

R Core Team. R: A Language and Environment for Statistical Computing.

## Supplementary Information

Supplementary Information 1.

Supplementary Information 2.

Supplementary Information 3.

Supplementary Information 4.

Supplementary Information 5.

Supplementary Information 6.

Supplementary Information 7.

Supplementary Information 8.

## Data Availability

Raw data from this study were deposited in the NCBI SRA (Sequence Read Archive) database numbers PRJNA673559.
