# Supplementary material for: Transcriptome analysis and phenotyping of walnut seedling roots under nitrogen stresses
Source: Sci Rep. 2022 Jul 14;12:12066. doi: 10.1038/s41598-022-14850-2 (PMC9283388; doi:10.1038/s41598-022-14850-2)
Supplement: Supplementary file 1 — Supplementary Information 1. [file 41598_2022_14850_MOESM1_ESM.docx]

**Methods for the Determination of Physiological Indexes**

The protein content was measured using the bicinchoninic acid (BCA) assay as described by Campion^[1]^. Briefly, 0–100 μg of protein standard was prepared and diluted to 100 μL using hydrogen peroxide, with 100 μL of pure water acting as a blank. Next, 2 mL of BCA working solution was added to tubes containing 100 μL of sample (three biological replicates per treatment), standard, or blank and mixed thoroughly. The tubes were incubated at 60°C for 15 min and then cooled to room temperature. The absorbance of the sample solutions was measured by spectrophotometry. The protein concentration in the samples was estimated based on the standard curve.

The total amino acid content was determined by ninhydrin assay^[2]^. Pure water was used as a blank, and glutamic acid was used as a standard. The samples were ground into powder with liquid nitrogen. Then, the blank, standard, and samples were transferred into centrifuge tubes containing 10 mL of distilled water and extracted by incubating in a boiling water bath for 45 min. After centrifugation (10,000 *g* for 10 min), the supernatants (1 mL each) were added to tubes containing 0.5 mL of 2% ninhydrin reagent and 0.5 mL of phosphate buffer (pH 8.0). The sample solutions were well mixed and allowed to react in a boiling water bath for 15 min. After cooling to room temperature, each tube was added to 8 mL of pure water and left to stand for 10 min.

The root NO_3_^-^ content was determined by the salicylic acid assay according to Liu^[3]^. The root samples from each treatment were dried in a ventilated oven at 80°C to constant weight. Dry roots (200 mg each) were ground into powder and transferred to tubes containing 10 mL of pure water, where they were extracted for 2.5 hours. At that time, the sample, blank (pure water), and NO_3_^-^ standard solutions (0.2 mL each) were transferred into 10% (mass ratio) salicylic acid and mixed in 96% sulfuric acid. The absorbance of the sample solutions was measured spectrophotometrically. The NO_3_^-^ concentration in the samples was estimated based on the standard curve. In accordance with the method by Prinsi^[4]^, five volumes (v/w) of 10 mM formic acid prepared in an ice bath were added to centrifuge tubes containing the samples, followed by homogenisation at 4°C for 15 min. Following centrifugation (14,000 *g* for 10 min at 4°C), the supernatants were filtered, and the NH_4_^+^ concentration was assayed by colourimetry.

The total nitrogen content was determined by the Kjeldahl method. The samples were added to digestion tubes containing 10 mL of concentrated sulfuric acid and digested until the sample solutions were clear. Thereafter, the digestion tubes were fixed on an automatic Kjeldahl nitrogen analyser (Hanon kll00, China) to determine the total nitrogen content of the roots.

The PRO concentration was measured by the method of Vieira^[5]^. Briefly, a 10-mL sample was diluted with pure water to a final volume of 200 mL. Then, a 1.0-mL aliquot was transferred to a tube to which 1 mL formic acid and 2 mL ninhydrin were added. The mixture was mixed well and incubated in a boiling water bath for 15 min. After cooling, the sample solution was added to 10 mL of *n*-butyl acetate and mixed thoroughly. The organic phase was filtered through filter paper containing anhydrous sodium sulfate, and the PRO concentration was measured spectrophotometrically.

The MDA concentration was measured with the thiobarbituric acid (TBA) assay as described by Castrejón and Yatsimirsky^[6]^. The root sample (0.3 g) was added to 2 mL of 0.05 mol/L phosphate buffer and ground into a homogenate. The homogenate was transferred to a tube, and the mortar was rinsed with 3 mL of 0.05 mol/L phosphate buffer. The extract was added to 5 mL of 0.5% TBA and mixed well. The tube was kept in a boiling water bath for 10 min and then immediately transferred to a cold water bath. After cooling, the solution was centrifuged at 3000 *g* for 15 min, and the supernatant was taken to measure the MDA concentration spectrophotometrically.

High-performance liquid chromatography was used to measure the levels of hormones (IAA, ABA, ZA, and GA3). In brief, approximately 0.1 g of root sample was added to 1 mL of precooled reagent I (methanol:water:acetic acid = 80:20:1), extracted overnight at 4°C, and centrifuged at 8000 *g* for 10 min. Next, the residue was extracted with 0.5 mL of reagent 1 (methanol:water:acetic acid = 80:20:1) for 2 hours and then centrifuged. The two supernatants were combined and blown with nitrogen gas at 40°C until there was no organic phase. After that, the sample was added to 0.5 mL of reagent II (petroleum ether), extracted, and decolourised three times at 60°C to 90°C. The upper ether phase was discarded, and the remainder was added to reagent III (saturated citric acid solution) to adjust the pH to 2.8. Then, the sample was extracted three times with reagent IV (ethyl acetate), the organic phases were combined, and the sample was blow-dried with nitrogen. The dry sample was added to 0.5 mL of reagent V (methanol), vortexed, shaken to dissolve, and passed through a syringe filter before use. Chromatographic analysis was completed on a RIGOL L3000 high-performance liquid chromatograph (RIGOL, China) equipped with a RIGOL C18 reversed-phase column (250 mm×4.6 mm, 5 μm; RIGOL) at an ultraviolet wavelength of 254 nm. For IAA, ABA, and ZA quantification, the following parameters were used: column temperature, 35°C; time, 40 min; flow rate, 0.8 mL/min; injection volume, 10 μL; mobile phase A: 1% aqueous acetic acid solution; mobile phase B: methanol (A:B = 6:4). For GA3 analysis, the parameters were as follows: column temperature, 30°C; time, 30 min; flow rate, 1 mL/min; injection volume, 10 μL; mobile phase A: 1% aqueous acetic acid solution; mobile phase B: methanol (A:B = 7.5:3.5).

**References**

[1] Campion, E.M., Loughran, S.T., Walls, D. Protein quantitation and analysis of purity. *Methods Mol. Biol*. **1485,** 225–255 (2016).

[2] Chen, Y., Fu, X., Mei, X., Zhou, Y., Cheng, S., Zeng, L., Dong, F., Yang, Z. Proteolysis of chloroplast proteins is responsible for accumulation of free amino acids in dark-treated tea (*Camellia sinensis*) leaves. *J. Proteom*. **157,** 10–17 (2017).

[3] Liu, G., Du, Q., Li, J. Interactive effects of nitrate-ammonium ratios and temperatures on growth, photosynthesis, and nitrogen metabolism of tomato seedlings. *Sci. Hortic*. **214,** 41–50 (2017).

[4] Prinsi, B., Negrini, N., Morgutti, S., Espen, L. Nitrogen starvation and nitrate or ammonium availability differently affect phenolic composition in green and purple basil. *Agronomy* **10,** 498 (2020).

[5] Vieira, S.M., Silva, T.M., Glória, M.B.A.. Influence of processing on the levels of amines and proline and on the physico-chemical characteristics of concentrated orange juice. *Food Chem*. **119,** 7–11 (2010).

[6] Castrejón, S.E., Yatsimirsky, A.K. Cyclodextrin enhanced fluorimetric determination of malonaldehyde by the thiobarbituric acid method. *Talanta*. **44,** 951–957 (1997).
