# Supplementary material for: Transcriptome analysis and phenotyping of walnut seedling roots under nitrogen stresses
Source: Sci Rep. 2022 Jul 14;12:12066. doi: 10.1038/s41598-022-14850-2 (PMC9283388; doi:10.1038/s41598-022-14850-2)
Supplement: Supplementary file 2 — Supplementary Information 2. [file 41598_2022_14850_MOESM2_ESM.docx]

**Supplementary Material**

| Gene ID | Forward primer sequence | Reverse primer sequence |
| --- | --- | --- |
| CL6197.Contig1_All | TCCAAGCCGCTATGAGAACC | TGGTCGAGGTAACGGAGGAA |
| CL7114.Contig1_All | AGACGGACGAAGCTGAGTTG | GTCGGAGGGTTAGCCAGTTC |
| Unigene8385_All | CGTGGCCTACTTCTCGATCC | GCAAATGGGTCAACCCACAC |
| CL8651.Contig7_All | GGAGCACCGTATCTACGCAA | GGGCACGTACTCAGAACCAT |
| Unigene19778_All | GGCACTACTGTGAAGCAACC | CCCAGAAGACTTGACACCGT |
| Unigene5814_All | CCTGAGGATGAGAATCGGCA | CTACTCGGCCACCAGCATTT |
| CL1426.Contig3_All | CACAGCCATGCCACTAAGGA | CTTGTTCAAGACCAAGCGGC |
| CL4305.Contig1_All | ACCCAGAGCAGAATGGTTGG | CTTAGGCACTTGCCACAAGC |
| CL5246.Contig2_All | TTAACAGGTGGAGTGGACGC | GCTCTGGTATCGTCGTCGTC |
| CL8824.Contig8_All | GAAACAACGGCGACGGAATC | GGCAAGAGTTTGCGAGTCAC |
| Unigene2028_All | CAGACCCGTCGCTATCTTCC | TTCTTGCTCGAGACCGAACC |
| Unigene27200_All | ATGGACCTTCCTCAACACGG | AGCCATCGAAGTTCCACTCG |
| CL415.Contig17_All | TCCTTGGCCCTGAGTCGATA | CAATCCCCTGCTGCAAACAC |
| Unigene12341_All | GACCCCAACTTCTGATGGCA | GAGTCGGAGCATGCAAACAC |
| CL1093.Contig1_All | GCCCTTCGACACGACAAAGT | TGGTCCCATCGTCTGGGATA |
| CL4119.Contig1_All | CCATGAAGGGGGTGGACATC | TATTGGCGAGGAGGAAGCTG |
| CL1179.Contig6_All | ATCTATGCCGGGCAATACCG | CCCGATGGGTCCACCTTATG |
| Unigene6821_All | GATCGGAAGCACCGGATCAA | TGATCGGGTGGAGGCTTAGA |
| Unigene27769_All | TGAGGGCAAGGCTGGCTAT | CCAGCTACCCCGTTATCACT |
| Unigene4961_All | CAGGGAATGTCGGTAGCTGT | TGGGTGCTGACTTGTCACTG |

**Table S1.** Primer sequence of qRT-PCR for root system of walnut seedling.

| Treatment | Protein content | Total amino acid content | Nitrate nitrogen content | Ammonium nitrogen content | Zeatin content | Indoleacetic acid content | Abscisic acid content | Gibberellic acid content |
| --- | --- | --- | --- | --- | --- | --- | --- | --- |
|  | mg/g | | mg/kg | | μg/g | | | |
| EL | 30.16±0.2590 b | 24.74±0.2515 b | 252.90±0.7130 c | 635.33±9.5523 c | 1.22±0.0014 c | 0.58±0.0179 b | 0.43±0.0008 b | 2.13±0.0102 b |
| L | 29.03±1.0815 b | 22.45±0.0677 c | 264.91±4.3101 b | 1062.42±10.9153 b | 1.40±0.0097 b | 0.43±0.0069 c | 0.43±0.0054 b | 1.17±0.0131 c |
| H | 56.97±1.1222 a | 27.92±0.0561 a | 281.32±3.0407 a | 1410.25±14.4471 a | 3.23±0.0269 a | 1.52±0.0197 a | 0.65±0.0078 a | 2.49±0.0125 a |

**Table S2.** Effects of nitrogen starvation and excess stress on the root morphology and physiology of walnut seedlings. The first column lists the different treatments (L = control, EL = nitrogen starvation, H = nitrogen excess), while the top row shows the indicators and the second row shows the indicator units. The data are the means ± SE. Different letters indicate statistical significance between the treatments tested by analysis of variance (P<0.05).

| Sample | RawReads | CleanReads | Q20(%) | Q30(%) | GC(%) | TotalMapped | MultipleMapped | UniquelyMapped |
| --- | --- | --- | --- | --- | --- | --- | --- | --- |
| EL1 | 74438410 | 69803704 | 97.48 | 89.56 | 43.67 | 62739569(89.88%) | 47751086(76.11%) | 14988483(23.89%) |
| EL2 | 69143101 | 64665682 | 97.37 | 89.22 | 43.55 | 57578323 (89.04%) | 44151058(76.68%) | 13427265(23.32%) |
| EL3 | 71858913 | 67937239 | 97.45 | 89.47 | 43.75 | 61109547(89.95%) | 46314925(75.79%) | 14794621(24.21%) |
| L1 | 69038244 | 65305313 | 97.41 | 89.29 | 43.99 | 58644171(89.80%) | 43789603(74.67%) | 14854569(25.33%) |
| L2 | 71638712 | 67465380 | 97.30 | 88.94 | 43.41 | 59990216(88.92%) | 45184631(75.32%) | 14805585(24.68%) |
| L3 | 69206016 | 64455967 | 97.32 | 89.10 | 43.79 | 57784774(89.65%) | 43627504(75.50%) | 14157270(24.50%) |
| H1 | 69237473 | 65986888 | 97.69 | 90.10 | 43.21 | 58352205(88.43%) | 44476050(76.22%) | 13876154(23.78%) |
| H2 | 69258445 | 65777172 | 97.50 | 89.55 | 43.54 | 57653692(87.65%) | 43136492(74.82%) | 14517200(25.18%) |
| H3 | 71428997 | 67979182 | 97.56 | 89.72 | 43.51 | 60664622(89.24%) | 46208243(76.17%) | 14456379(23.83%) |

**Table S3.** RNA sequencing and read mapping data from the roots of walnut seedlings treated with different nitrogen levels. Q20, percentage of bases with a quality score >20; Q30, percentage of bases with a quality score >30; GC, guanine+cytosine content.
