# Supplementary figures and images for: Transcriptome analysis and phenotyping of walnut seedling roots under nitrogen stresses
Source: Sci Rep. 2022 Jul 14;12:12066. doi: 10.1038/s41598-022-14850-2 (PMC9283388; doi:10.1038/s41598-022-14850-2)

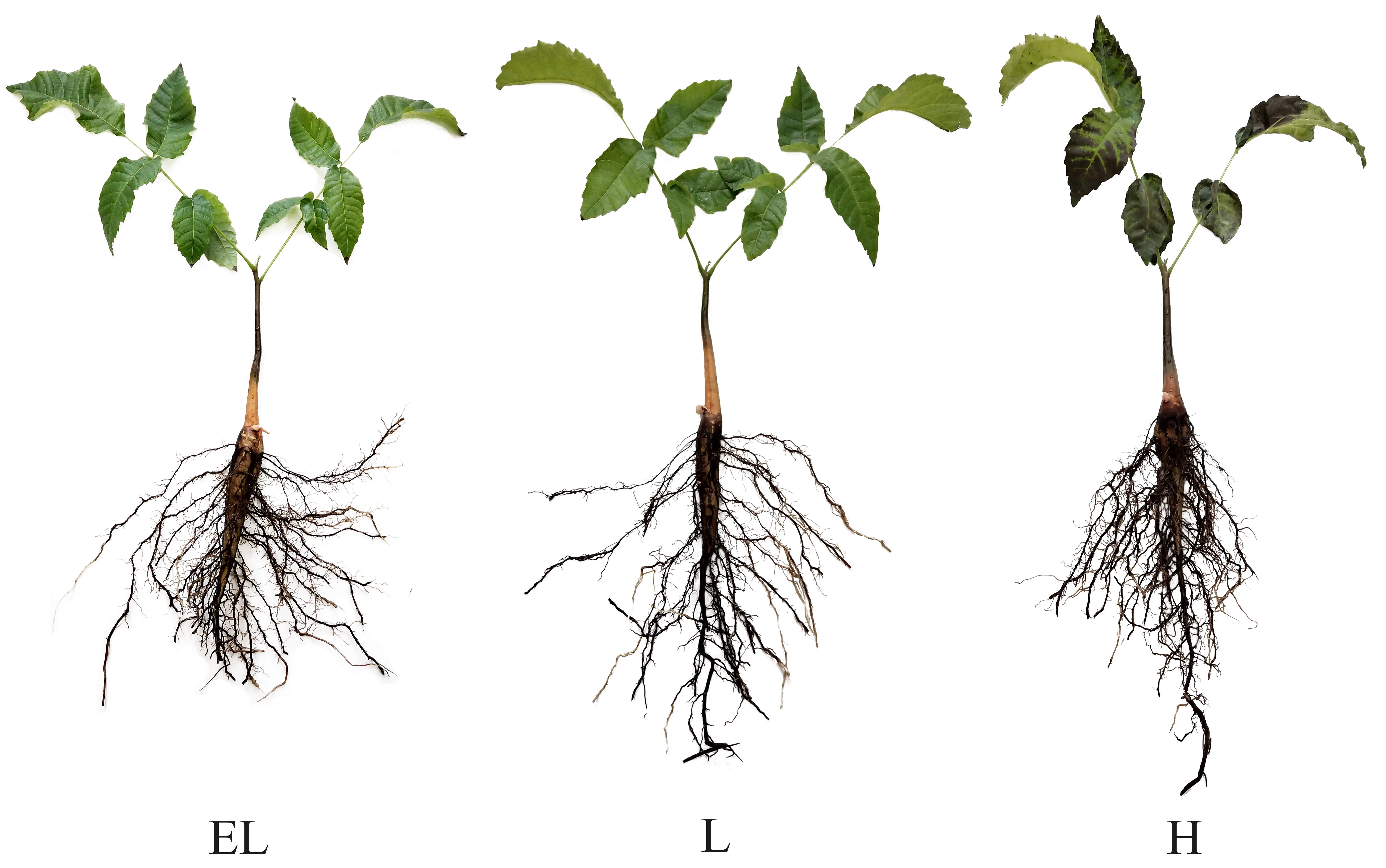

Supplement: Supplementary file 8 — Supplementary Information 8. [file 41598_2022_14850_MOESM8_ESM.jpg]
